# Mining Database for the Expression and Clinical Significance of NF-*κ*B Family in Hepatocellular Carcinoma

**DOI:** 10.1155/2020/2572048

**Published:** 2020-08-17

**Authors:** Xu Chen, Yufan Zhou, Zhecheng Li, Zhiming Wang

**Affiliations:** Department of General Surgery, Xiangya Hospital, Central South University, Changsha 410008, China

## Abstract

**Background:**

Hepatocellular carcinoma (HCC) is one of the deadliest diseases affecting humans. Its incidence has been increasing over the last decade. It is characterized by poor prognosis as well as lack of therapeutic regimens for patients in the advanced stages. It is therefore important to develop effective biomarkers for diagnosis, prognosis, and immunotherapy of HCC. Research suggests that the NF-*κ*B family plays vital roles in immune response, inflammation, tumorigenesis, and the progress of malignancy in various cancers. However, its role in HCC remains unidentified. *Methodology*. The expression and clinical significance of the NF-*κ*B family in HCC were analyzed using several bioinformatics tools including UALCAN, The Human Protein Atlas, GEPIA, GSCALite, David, GeneMANIA, and TIMER.

**Results:**

The mRNA expression levels of RelA, RelB, NF-*κ*B1, and NF-*κ*B2 were significantly elevated in HCC. The mRNA levels of RelB and NF-*κ*B2 were significantly upregulated in HCC tissues compared to normal liver tissues in subgroup analyses based on patient's race, gender, age, weight, tumor grade, cancer stage, and nodal metastasis status. Moreover, HCC patients with elevated levels of RelB and NF-*κ*B2 had a worse overall survival and disease-free survival. Methylation downregulated the expressions of RelA, RelB, and NF-*κ*B1 in HCC. NF-*κ*B family was also significantly involved in various hallmark cancer-related pathways such as the apoptosis, EMT, RTK, and cell cycle pathways. Similarly, the expression of RelB and NF-*κ*B2 was positively correlated with the abundance of immune cells and the expression of immune biomarkers. Several kinase and miRNA targets of RelB and NF-*κ*B2 were also identified.

**Conclusion:**

RelB and NF-*κ*B2 are potential biomarkers for the diagnosis, prognosis, and immunotherapy of HCC.

## 1. Introduction

Hepatocellular carcinoma (HCC) is one of the deadliest diseases that affect humans and the second cause of cancer-related deaths globally. It is the fifth most common malignancy in men and the ninth in women [1]. Moreover, its incidence has been increasing in the past ten years [2]. The main risk factors for HCC are cirrhosis, chronic viral infection, and alcoholic liver disease [3]. The prognosis of HCC patients is poor. It is usually diagnosed at an advanced stage and lacks therapeutic regimens at this stage. Considerably, there is a need for effective novel biomarkers for diagnosis, prognosis, and immunotherapy of HCC.

The transcription factor nuclear factor-kappa B (NF-*κ*B), identified in the 1980s, functions by binding to the enhancer element of the immunoglobulin kappa light chain of activated B cells [4]. In the immune cell function, NF-*κ*B control is mediated by the canonical and noncanonical NF-*κ*B signaling pathways. Here, the critical terminal components of the NF-*κ*B signaling pathway are the I*κ*B protein (inhibitor) and IKK complex (activator) [5]. The NF-*κ*B family has 5 distinct subunits that include RelA, RelB, Rel, NF-*κ*B1, and NF-*κ*B2 [6]. Growing evidence highlights the significance of the NF-*κ*B family in immune response and inflammation as well as in tumorigenesis and progress of malignancy [4, 7]. Moreover, the NF-*κ*B family has been postulated as biomarkers for various cancers [8]. For example, the NF-*κ*B family has been reported to play a key role in tumor migration and as a potential therapeutic target in colorectal cancer [9]. Similarly, RelA serves as a prognostic biomarker in chronic lymphocytic leukemia [10]. However, the role of the NF-*κ*B family in HCC remains unclear.

Herein, the expression and clinical significance of the NF-*κ*B family were explored using various bioinformatics tools.

## 2. Materials and Methods

### 2.1. UALCAN

UALCAN is a comprehensive, user-friendly, and interactive web resource for analyzing The Cancer Genome Atlas (TCGA) data [10]. TCGA is a landmark cancer genomics program containing over 20,000 molecularly characterized primary cancers and matched normal samples spanning up to 33 cancer types. In this study, NF-*κ*B family was submitted to UALCAN and their mRNA expression in HCC was explored using TCGA HCC samples (*N* = 442). Moreover, the association between the NF-*κ*B family expression and the clinicopathological parameters was evaluated. *P* values less than 0.05 (*P* < 0.05) indicated that there were significant differences.

### 2.2. The Human Protein Atlas

The Human Protein Atlas is a Swedish-based program initiated in 2003 which maps all the human proteins in cells, tissues, and organs [8]. All the NF-*κ*B family members were submitted to The Human Protein Atlas and their protein expression in HCC was explored.

### 2.3. GEPIA

GEPIA is a newly developed interactive web server used to analyze RNA sequencing expression data via a standard processing pipeline. It contains 9,736 tumors and 8,587 normal samples data from the TCGA and the GTEx projects [11]. All the NF-*κ*B family members were submitted to the GEPIA and their correlated genes and prognostic value in HCC were explored using TCGA HCC samples (*N* = 442). The median level of the NF-*κ*B family group cutoff was determined through the Kaplan–Meier analysis method. The “Similar Genes” module was used to explore the top 10 significant genes correlated with NF-*κ*B family in HCC. The statistical differences associated with *P* < 0.05 were considered significant. The hazard ratio (HR) was calculated according to the Cox PH model.

### 2.4. GSCALite

GSCALite is a web-based analysis platform used for gene set cancer analysis. This includes methylation analysis, cancer pathway analysis, and drug analysis, among other analyses [12]. In GSCALite, the HCC genomics data from TCGA and the normal tissue data from The Genotype-Tissue Expression (GTEx) project were integrated to build a comprehensive public resource to study tissue-specific gene expression and regulation. The GTEx samples were collected from 54 nondiseased tissue sites across nearly 1000 individuals. All the NF-*κ*B family members were submitted to the GSCALite website to analyze the methylation of the NF-*κ*B family in HCC using the TCGA HCC samples (*N* = 442). Moreover, the role of NF-*κ*B family in cancer pathway activity and drug sensitivity, as well as NF-*κ*B family associated miRNA regulation network, was explored. *P* values or FDR less than 0.05 were considered as statistically significant.

### 2.5. Enrichment Analysis

David and GeneMANIA are bioinformatics portals which help individual researchers to gain more insights on the function of genes [13]. For enrichment analysis, all the members of the NF-*κ*B family were submitted to the David for Gene Ontology (GO) and Kyoto Encyclopedia of Genes and Genomes (KEGG) pathways. Furthermore, all the NF-*κ*B family members were submitted to the GeneMANIA and a protein-protein interaction (PPI) network was constructed to reveal their potential functions.

### 2.6. TIMER

TIMER is a web resource for systematic evaluation of the clinical impact of different immune cells in 23 cancer types from TCGA [11]. All the members of the NF-*κ*B family were submitted to the TIMER website and their correlation with immune cells and immune biomarkers in HCC was explored using TCGA HCC samples (*N* = 442). These immune biomarkers have been extensively encountered in previous studies [14–16].

## 3. Results

### 3.1. Profiles of NF-*κ*B Subunit Expression in HCC

**Figure 1:**
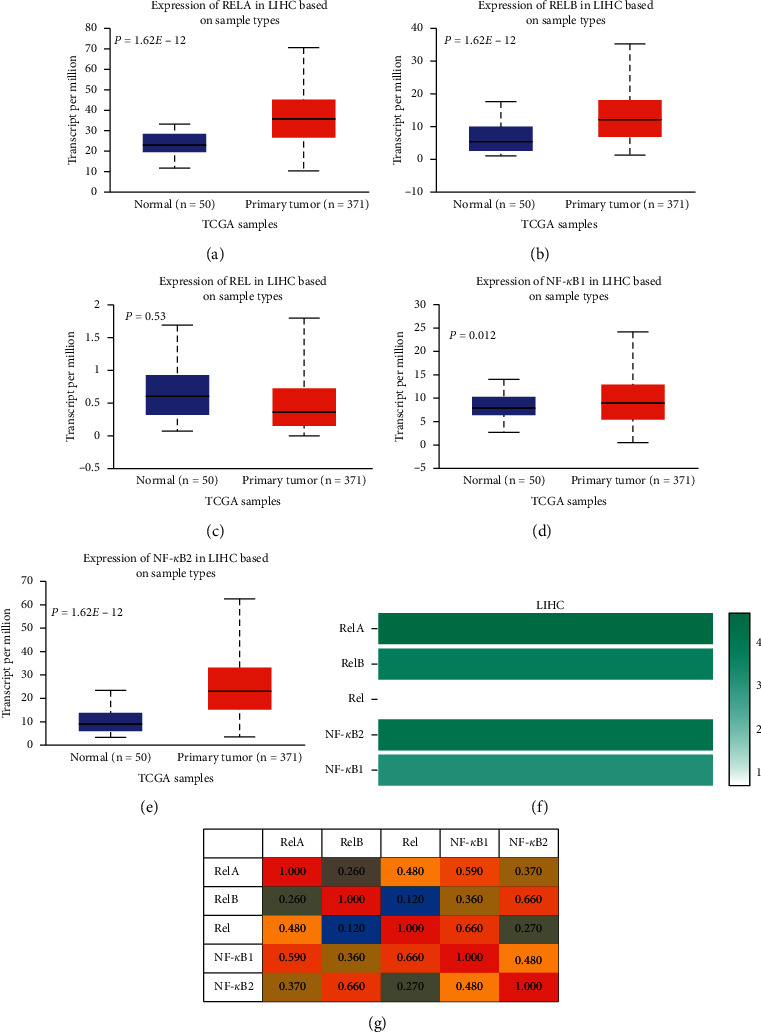
Expression profile of NF-*κ*B family in HCC. (a)–(e) The mRNA levels of NF-*κ*B family members in HCC tissues and normal tissues (UALCAN). (f) The relative mRNA level of each number of the NF-*κ*B family in HCC tissues (GEPIA). (g) The correlation of each number of the NF-*κ*B family in HCC tissues (GEPIA).

**Figure 2:**
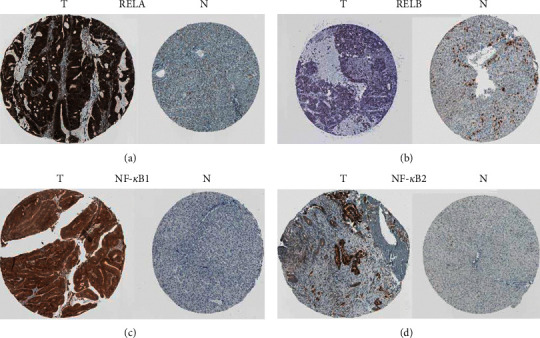
The expression of NF-*κ*B family at protein level in HCC (The Human Protein Atlas). (a)–(d) Low protein expression of RELA, RELB, NF-*κ*B1, and NF-*κ*B2 in normal liver tissues. Medium protein expression of RELB (b) and high protein expression of RELA (a), NF-*κ*B1 (c), and NF-*κ*B2 (d) in HCC tissues. T: tumor tissues, N: normal tissues.

HCC tissues exhibited significantly higher levels of RelA (*P* = 1.62^*E* − 12)^, RelB (*P* = 1.62^*E* − 12)^, NF-*κ*B1 (*P* = 0.012), and NF-*κ*B2 (*P* = 1.62^*E* − 12^) expression (Figures 1(a), 1(b), 1(d), and 1(e)) than normal liver tissues. Particularly, RelA had the highest, whereas Rel recorded the lowest, mRNA levels in HCC tissues (Figure 1(f)). Correlation analysis revealed a medium association among the NF-*κ*B family subunits in HCC tissues (Figure 1(g)). Furthermore, analysis of the dysregulated NF-*κ*B family subunits (RelA, RelB, NF-*κ*B1, and NF-*κ*B2), using tissues Atlas, revealed higher protein levels in HCC, relative to specimens from normal controls (Figure 2). Overall, these findings strongly affirmed the significance of NF-*κ*B family in HCC.

### 3.2. Diagnostic and Prognostic Value of NF-*κ*B Family in HCC

**Figure 3:**
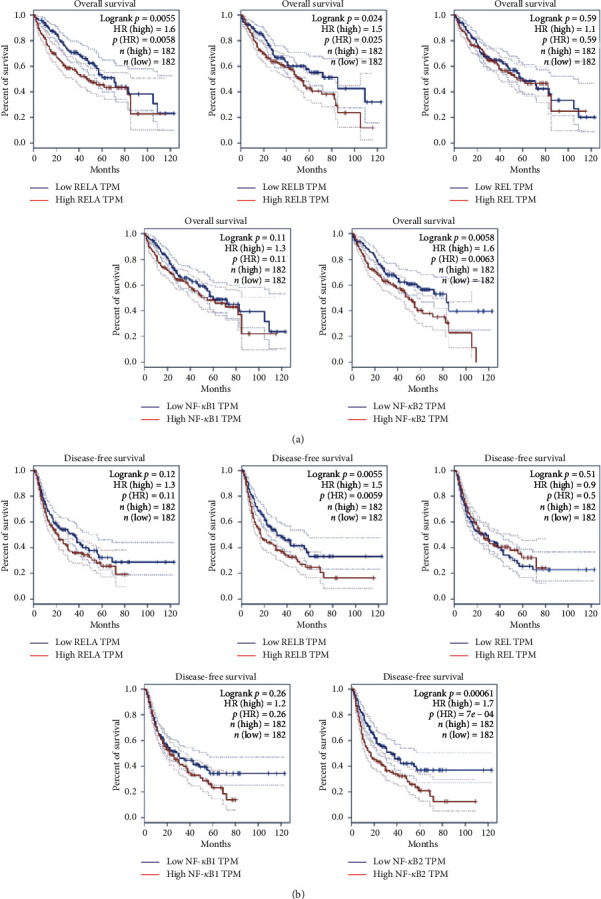
The prognostic value of NF-*κ*B family in HCC (GEPIA). (a) High expression of RelA, RelB, and NF-*κ*B2 correlated with worse overall survival of HCC patients. (b) High expression of RelB and NF-*κ*B2 correlated with worse disease-free survival of HCC patients. HR: hazard ratio.

Overall survival analysis revealed that HCC patients with high levels of RelA (HR = 1.6, *P* = 0.0055), RelB (HR = 1.5, *P* = 0.024), and NF-*κ*B2 (HR = 1.6, *P* = 0.0058) had a poor overall survival rates. Conversely, levels of Rel and NF-*κ*B1 expression had no significant effect on overall survival of HCC patients (Figure 3(a)). On the other hand, results from disease-free survival analysis showed that HCC patients with high levels of RelB (HR = 1.5, *P* = 0.0055) and NF-*κ*B2 (HR = 1.7, *P* = 0.00061) had poor disease-free survival, whereas RelA, Rel, and NF-*κ*B1 expression had no significant effect on disease-free survival of HCC patients (Figure 3(b)). These results suggested that RelB and NF-*κ*B2 could be potential prognostic biomarkers for HCC.

**Figure 4:**
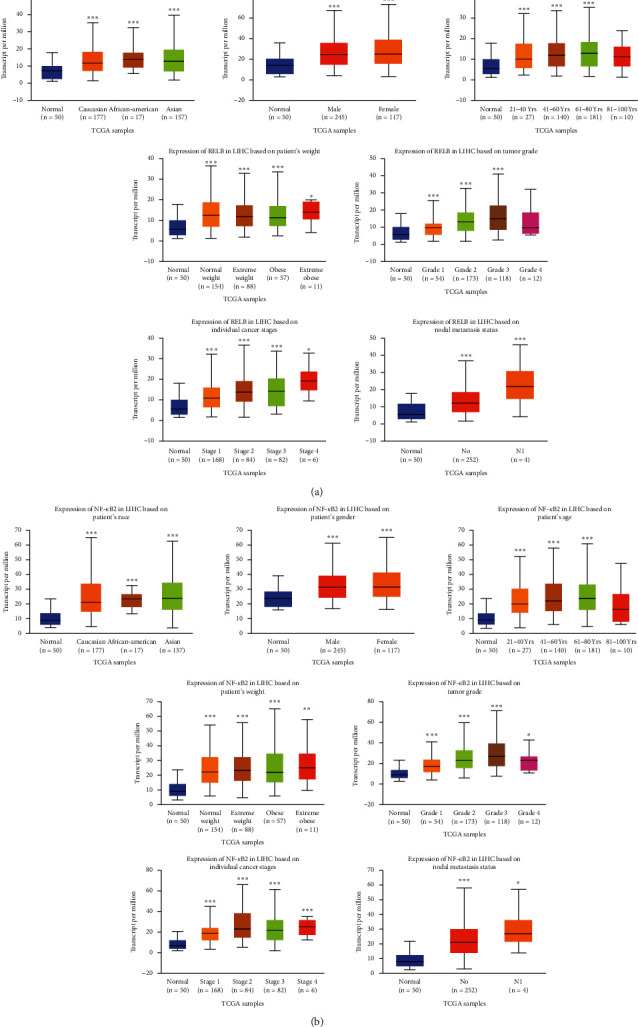
The expression of RelB and NF-*κ*B2 in HCC in subgroup analyses (UALCAN). Subgroup analyses were performed based on patients' race, gender, age, weight, tumor grade, individual cancer stages, and nodal metastasis status. ^*∗*^*P* < 0.05, ^*∗∗*^*P* < 0.01, ^*∗∗∗*^*P* < 0.001.

A correlation between RelB and NF-*κ*B2 expression with clinicopathological parameters, including a patient's race, gender, age, weight, tumor grade, cancer stage, and nodal metastasis status, revealed significant upregulation of RelB and NF-*κ*B2 in HCC, relative to normal liver tissues (Figure 4). These results suggested that RelB and NF-*κ*B2 may be involved in tumor invasiveness during HCC development.

### 3.3. NF-*κ*B Family Is Associated with Cancer Hallmarks in HCC

**Figure 5:**
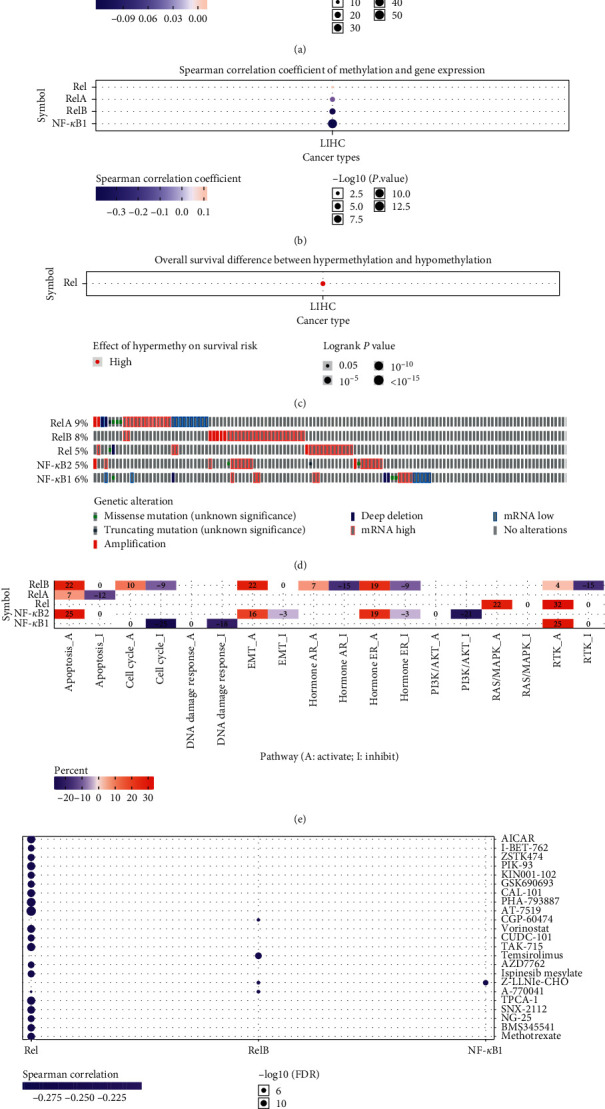
The association between NF-*κ*B family and cancer hallmarks (GSCALite). (a)–(c) Association between NF-*κ*B family and methylation. (d) Summary of genetic alterations of NF-*κ*B family in HCC. (e) The role of NF-*κ*B family in cancer-related pathways. (f) The correlation between NF-*κ*B family and drug sensitivity. *P* value or FDR <0.05 was considered as significant.

To evaluate the potential effects of disrupting the subunits in HCC patients, we correlated expression profiles of NF-*κ*B family subunits with methylation. The results revealed significantly lower levels of Rel, RelB, and NF-*κ*B2 methylation, whereas those of NF-*κ*B1 were significantly upregulated in HCC, relative to normal tissues (Figure 5(a)). In addition, methylation mediated downregulation of RelA, RelB, and NF-*κ*B1 but led to Rel upregulation in HCC tissues (Figure 5(b)). Moreover, HCC patients who exhibited hypermethylation had a better overall survival (Figure 5(c)). Generally, genetic alterations drive tumorigenesis and progression of cancer cells. In the present study, we found alteration of RelA, RelB, Rel, NF-*κ*B1, and NF-*κ*B2 in 9, 8, 5, 6, and 5% of the queried HCC samples, respectively. These alterations included missense and truncating mutations, amplification, deep deletion, and high and low mRNA levels (Figure 5(d)). In addition, we explored the role played by members of the NF-*κ*B family in cancer hallmark pathways, including TSC/mTOR, RTK, RAS/MAPK, PI3K/AKT, hormone ER, hormone AR, EMT, DNA damage response, cell cycle, and apoptosis. The results showed that the NF-*κ*B family was significantly involved in the aforementioned pathways. Specifically, the subunits were associated with activation of the apoptosis, EMT, and RTK pathways, as well as the inhibition of the cell cycle pathway (Figure 5(e)). A correlation between expression of members of the NF-*κ*B family and drug sensitivity was also evaluated to identify potential therapeutic targets in HCC tissues. Summarily, low expression of Rel and RelB was associated with drug sensitivity (Figure 5(f)). Further analysis revealed that 20 miRNAs potentially regulated RelA, whereas 12 miRNAs potentially regulated NF-*κ*B (Supplementary Figure 1).

### 3.4. Enrichment Analysis of NF-*κ*B Family in HCC

**Figure 6:**
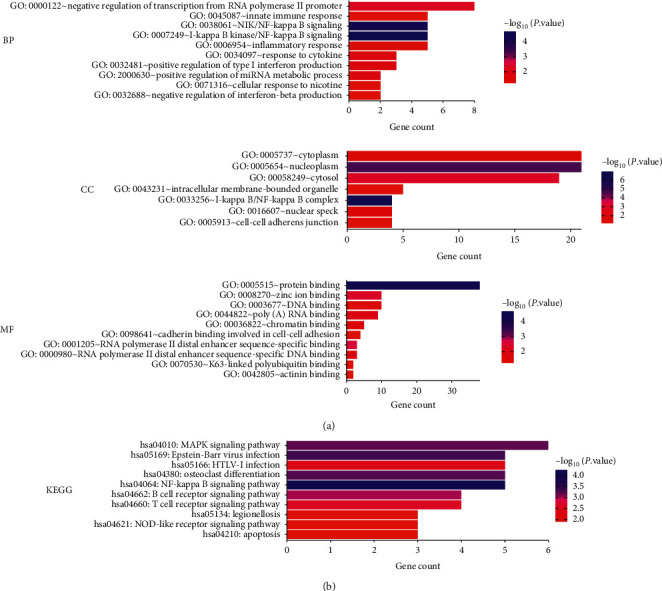
Enrichment of NF-*κ*B family in HCC (DAVID). (a) Bar plot of GO enrichment in cellular component (BP) terms, biological process (CC) terms, and molecular function (MF) terms. (b) Bar plot of Kyoto Encyclopedia of Genes and Genomes (KEGG) enriched terms.

**Figure 7:**
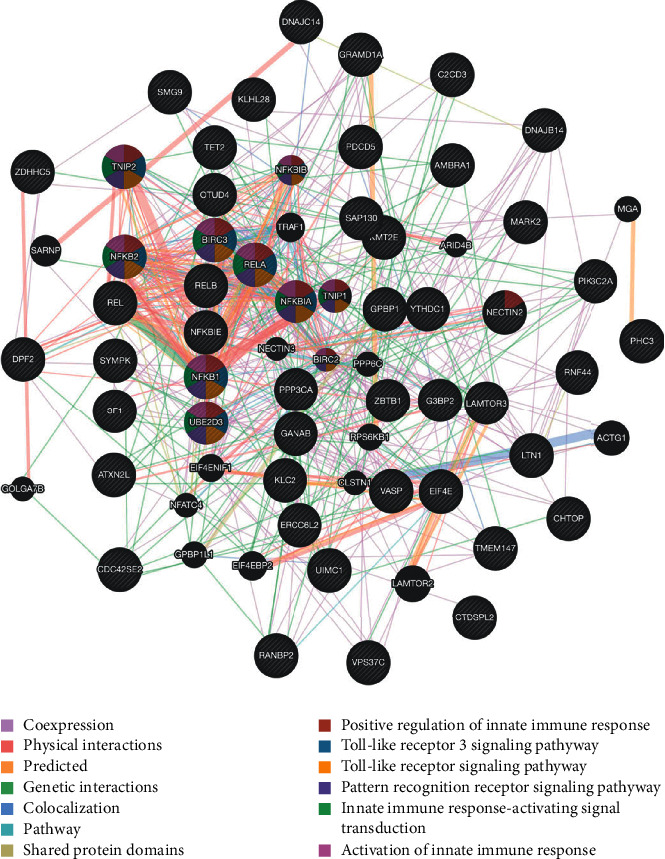
Protein-protein interaction (PPI) network of NF-*κ*B family (GeneMANIA). Different colors in the network edge indicate the bioinformatics methods applied: coexpression, website prediction, pathway, physical interactions, and colocalization. Network colored nodes indicate the biological functions of specific sets of enriched genes.

To evaluate the potential effects of NF-*κ*B family subunits in HCC patients, we performed an enrichment analysis and then determined the ten most significant genes associated with each NF-*κ*B family subunit. A summary of the results is provided in Supplementary Table 1. The NF-*κ*B family subunits and the most significant genes were then submitted to David and GeneMANIA for enrichment analysis. Summarily, GO analysis results revealed that the NF-*κ*B family subunits were mainly involved in innate immune response, NIK/NF-kappa B signaling, inflammatory response, protein binding, DNA and poly(A) RNA binding, and cell adhesion (Figure 6(a)). In addition, the results from KEGG pathways analysis revealed that the NF-*κ*B family subunits were mainly involved in the MAPK, NF-kappa B, as well as B and T cell receptor, NOD-like receptor signaling pathways, and apoptosis (Figure 6(b)). Similarly, the PPI network indicated that NF-*κ*B family subunits are also involved in innate immune response, toll-like receptor, and pattern recognition receptor signaling pathways (Figure 7).

### 3.5. NF-*κ*B Family Subunits Are Associated with Immune Infiltration in HCC

**Figure 8:**
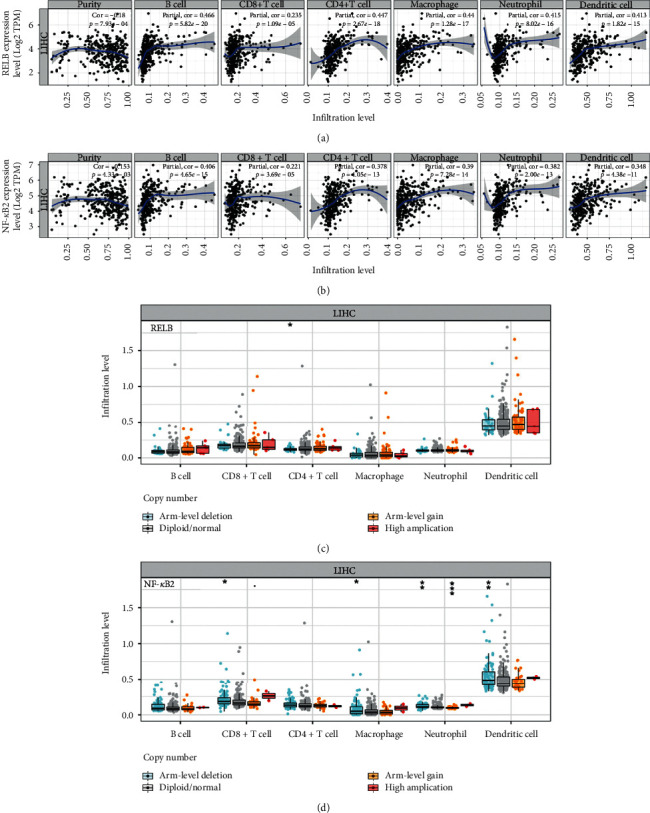
Association of immune infiltration with RelB and NF-*κ*B2 in HCC (TIMER). (a)-(b) Relationship of RelB and NF-*κ*B2 with the abundance of different immune cells in HCC. (c)-(d) Association of copy number alteration of RelB and NF-*κ*B2 with immune cell infiltration in HCC.

The role of NF-*κ*B family subunits in immune infiltration in HCC was further explored, based on the results from enrichment analysis which had earlier suggested that the NF-*κ*B family is involved in immune response. For this analysis, we selected RelB and NF-*κ*B2, due to their high significance in HCC progression. The results revealed a strong positive correlation between RelB expression with immune infiltration level of B (Cor = 0.466, *P* = 5.82^*e* − 20^), CD8+ T (Cor = 0.235, *P* = 1.09^*e* − 05^), and CD4+ T cells (Cor = 0.447, *P* = 2.^67*e* − 18^), as well as macrophages (Cor = 0.44, *P* = 1.28^*e* − 17^), neutrophils (Cor = 0.415, *P* = 8.02^*e* − 16^), and dendritic cells (Cor = 0.413, *P* = 1.82^*e* − 15^) (Figure 8(a)). Similarly, NF-*κ*B2 expression had a strong positive correlation with immune infiltration levels of B (Cor = 0.406, *P* = 4.65^*e* − 15^), CD8+ T (Cor = 0.221, *P* = 3.69^*e* − 05^), and CD4+ T cells (Cor = 0.378, *P* = 4.05^*e* − 13^), as well as macrophages (Cor = 0.39, *P* = 7.28^*e* − 14^), neutrophils (Cor = 0.382, *P* = 2.00^*e* − 13^), and dendritic cells (Cor = 0.348, *P* = 4.38^*e* − 11^) (Figure 8(b)). Conversely, RelB-mediated copy number alteration had no significant effect on immune infiltration (Figure 8(c)), although that of NF-*κ*B2 partly promoted immune infiltration (Figure 8(d)). Moreover, RelB and NF-*κ*B2 expression had a significant positive correlation with a majority of the immune biomarker sets or immune checkpoint inhibitors in HCC (Supplementary Tables 2 and 3). These results strongly suggested that RelB and NF-*κ*B2 may be potential immune checkpoint inhibitors for HCC.

### 3.6. Kinase and miRNA Targets of RelB and NF-*κ*B2

We determined kinase and miRNA targets in RelB and NF-*κ*B2, owing to these subunits' significance in HCC. The top 5 most significant kinase targets for RelB in HCC were kinase SYK, LCK, PRKCG, LYN, and ROCK1, whereas its miRNA targets were ATAAGCT (MIR-21), TAATGTG (MIR-323), ATGTTAA (MIR-302C), TAGGTCA (MIR-192 and MIR-215), and TCTATGA (MIR-376A and MIR-376B) (Supplementary Table 4). On the other hand, the top 5 most significant kinase targets for NF-*κ*B2 in HCC were CHUK, PPKDC, IKBKB, ROCK1, and PPKCA, whereas its top 5 most significant miRNA targets were ACACTGG (MIR-199A and MIR-199B), GCATTTG (MIR-105), TAGCTTT (MIR-9), TGCACTT (MIR-519C, MIR-519B, and MIR-519A), and GCTTGAA (MIR-498) (Supplementary Table 5). These results suggested that RelB and NF-*κ*B2 potentially exert various functions in HCC via these targets.

## 4. Discussion

The NF-*κ*B family regulates various biological processes such as inflammation, immune response, cellular proliferation, and apoptosis [17]. Dysregulation of the NF-*κ*B family, therefore, leads to a series of diseases ranging from cancers to inflammatory and immune disorders. Several studies have explored the potential role of NF-*κ*B family subunits in the diagnosis, prognosis, and therapy of cancers [18]. Herein, the expression and clinical significance of NF-*κ*B family subunits in HCC were explored through data mining from various databases using bioinformatics tools.

Our analyses revealed that RelA, RelB, NF-*κ*B1, and NF-*κ*B2 were markedly higher in HCC tissues than in normal liver tissues. Moreover, RelB and NF-*κ*B2 showed the potential to be diagnostic and prognostic biomarkers of HCC. Currently, some NF-*κ*B family subunits have been reported to be biomarkers in other types of cancers. For example, RelB is involved in the regulation of cell cycle and can predict good prognosis in glioma [19]. Similarly, high REL levels have been shown to predict response to immunochemotherapy in follicular lymphoma [20]. It is therefore likely that NF-*κ*B family subunits may influence the tumorigenesis and progression of HCC.

Changes in the expression of NF-*κ*B family subunits affect activities of several cancer-related pathways. For instance, it has been reported to inhibit the cell cycle pathway and activate apoptosis, EMT, and RTK pathways. This has been demonstrated in many studies [21–23]. In a study by Pires et al., NF-*κ*B family-regulated expression of genes involved in EMT process in breast cancer [24]. NF-*κ*B family-regulated EMT process has been linked to tumorigenesis, growth, metastasis, drug resistance, and progression of cancers [25–28]. A significant correlation between NF-*κ*B family and EMT pathway has been reported in HCC tissues. HAX-1 promoted HCC metastasis by enhancing EMT process via the NF-*κ*B pathway [29]. Elsewhere, NKILA inhibited tumor metastasis by suppressing the NF-*κ*B/Slug mediated ETM pathway in HCC [30]. Herein, some members of the NF-*κ*B family were found to influence tumor stage, grade, metastasis, and the overall survival of HCC patients. The NF-*κ*B family subunits influenced the aggressiveness of HCC cells by regulating EMT pathway.

Here, we demonstrate a correlation between NF-*κ*B family subunits and immune infiltration. The expression of RelB and NF-*κ*B2 was positively correlated with infiltration of several immune cells (B cells, CD8+ T cells, CD4+ T cells, macrophages, neutrophils, and dendritic cells) and the expression of immune biomarkers such as PD-1 and CTLA4. Some of these immune cells or immune biomarkers have been reported to be biomarkers or immunotherapeutic targets of HCC. Increased CD4(+)CD25(+)FoxP3(+) Treg may impair the effector function of CD8(+) T cells, promote HCC progression, and serve as a potential prognostic marker and a therapeutic target for HCC [31]. Dendritic cells are an attractive target for therapeutic manipulation in HCC [32]. Upregulation of PD-L1 is strongly associated with poor survival in HCC patients. As such, it may act as a potential immunotherapeutic target for HCC [33]. Herein, RelB and NF-*κ*B2 were found to be potential immunotherapeutic targets for HCC. However, further studies involving the use of animal models should be performed to verify these results.

Several kinase targets of RelB and NF-*κ*B2 in HCC were also identified. Among them, SYK, LCK, and LYN are involved in regulation of genomic stability, mitosis, and the cell cycle [34, 35]. As such, RelB and NF-*κ*B2 may regulate DNA damage response and cell cycle progression via these kinases [36]. Several miRNAs associated with RelB and NF-*κ*B2 were also identified. They included miR-21, miR-9, and miR-105. These miRNAs regulate cell proliferation and invasion in HCC [37, 38]. Moreover, these miRNAs have been used as diagnostic and prognostic markers of HCC [39]. These findings show that the NF-*κ*B family may play a vital role in tumorigenesis and progression of HCC via these kinases and miRNAs.

## 5. Conclusion

This study reveals the expression profile and clinical significance of NF-*κ*B family in HCC. The results demonstrate that RelB and NF-*κ*B2 are potential biomarkers for the diagnosis, prognosis, and immunotherapy of HCC.

## Data Availability

All the data and results generated during the study are available from the corresponding author on reasonable request.

## Supplementary Materials

Supplementary Materials Supplementary Figure 1: NF-*κ*B family associated miRNA regulation network showing the regulation of miRNAs to NF-*κ*B family. Supplementary Table 1: the top 10 significant genes correlated with NF-*κ*B family in HCC (GEPIA). Supplementary Table 2: correlation analysis between RelB and gene biomarkers of immune cells in HCC (TIMER). Supplementary Table 3: correlation analysis between NF-*κ*B2 and gene biomarkers of immune cells in HCC (TIMER). Supplementary Table 4: the Kinase and miRNA-target networks of RelB in HCC (LinkedOmics). Supplementary Table 5: the Kinase and miRNA-target networks of NF-*κ*B2 in HCC (LinkedOmics).
