# Supplementary material for: Mining Database for the Expression and Clinical Significance of NF-κB Family in Hepatocellular Carcinoma
Source: J Oncol. 2020 Aug 17;2020:2572048. doi: 10.1155/2020/2572048 (PMC7448221; doi:10.1155/2020/2572048)
Supplement: Supplementary Materials — Supplementary Figure 1: NF-κB family associated miRNA regulation network showing the regulation of miRNAs to NF-κB family. Supplementary Table 1: the top 10 significant genes correlated with NF-κB family in HCC (GEPIA). Supplementary Table 2: correlation analysis between RelB and gene biomarkers of immune cells in HCC (TIMER). Supplementary Table 3: correlation analysis between NF-κB2 and gene biomarkers of immune cells in HCC (TIMER). Supplementary Table 4: the Kinase and miRNA-target networks of RelB in HCC (LinkedOmics). Supplementary Table 5: the Kinase and miRNA-target networks of NF-κB2 in HCC (LinkedOmics). [file 2572048.f1.docx]

**
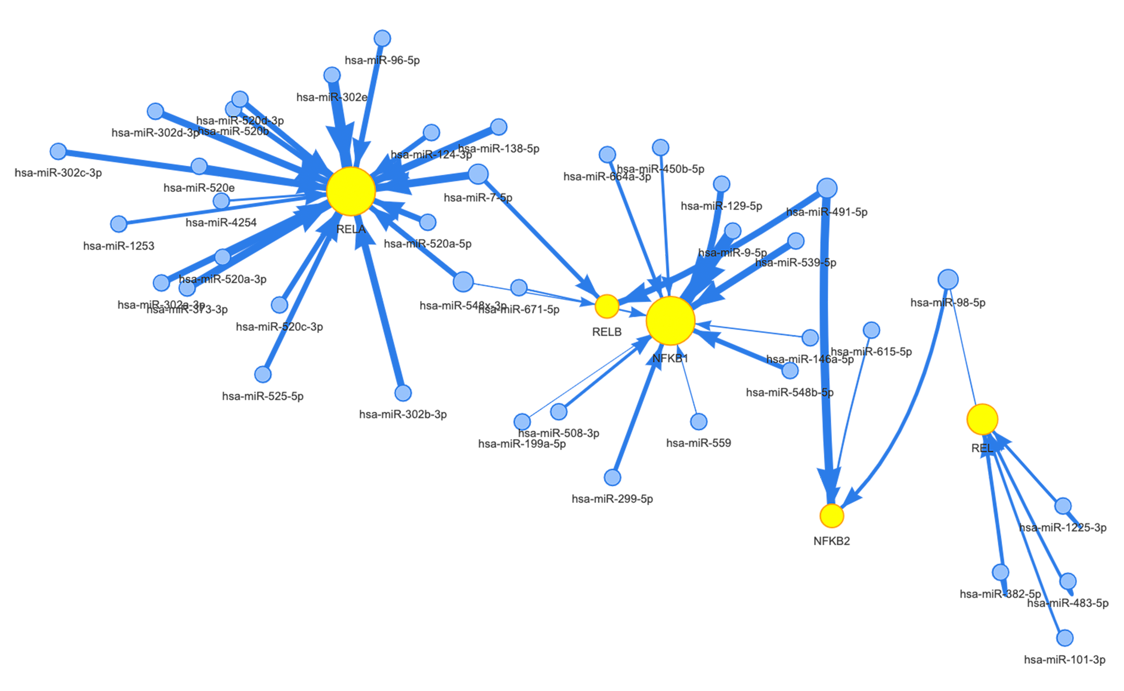
**

**Supplementary Figure 1. NF-κB family associated miRNA regulation network showing the regulation of miRNAs to NF-κB family**

**Supplementary Table 1. The top 10 significant genes correlated with NF-κB family in HCC (GEPIA).**

| **NF-κB family** | **Correlated genes** |
| --- | --- |
| RelA  RelB  Rel  NF-κB1  NF-κB2 | ZDHHC5, DPF2, C2CD3, MARK2, VPS37C, AMBRA1, SF1, GANAB, DNAJC14, SAP130  NF-κB2, GRAMD1A, PDCD5, NFKBIE, VASP, SYMPK, AC002398.13, PVRL2, TMEM147, SMG9  KLHL28, NONOP2, PHC3, RP11-159G9.5, LTN1, ERCC6L2, PIK3C2A, RANBP2, KMT2E, CTDSPL2  RELB, CDC42SE2, KLC2, CHTOP, ATXN2L, UIMC1, RNF44, NFKBIE, GPBP1, BIRC3  EIF4E, YTHDC1, TET2, DNAJB14, ZBTB1, UBE2D3, LAMTOR3, PPP3CA, G3BP2, OTUD4 |

**Supplementary Table 2. Correlation analysis between RelB and gene biomarkers of immune cells in HCC (TIMER).**

| **Immune cells** | **Biomarkers** | Cor | P-value | Cor | | P-value |
| --- | --- | --- | --- | --- | --- | --- |
| CD8+ T cell | CD8A  CD8B | 0.313  0.297 | ***  *** | 0.253  0.248 | ***  *** | |
| T cell (general) | CD3D  CD3E  CD2 | 0.441  0.42  0.427 | ***  ***  *** | 0.401  0.38  0.39 | ***  ***  *** | |
| B cell | CD19  CD79A | 0.304  0.301 | ***  *** | 0.252  0.238 | ***  *** | |
| Monocyte | CD86  CD115(CSF1R) | 0.491  0.446 | ***  *** | 0.463  0.41 | ***  *** | |
| TAM | CCL2  CD68  IL10 | 0.41  0.343  0.359 | ***  ***  *** | 0.366  0.284  0.3 | ***  ***  *** | |
| M1 Macrophage | INOS (NOS2)  IRF5  COX2(PTGS2) | 0.071  0.388  0.372 | 0.171  ***  *** | 0.048  0.384  0.324 | 0.375  ***  *** | |
| M2 Macrophage | CD163  VSIG4  MS4A4A | 0.244  0.359  0.332 | ***  ***  *** | 0.178  0.32  0.285 | ***  ***  *** | |
| Neutrophils | CD66b (CEACAM8)  CD11b (ITGAM)  CCR7 | 0.021  0.544  0.344 | 0.683  ***  *** | 0.012  0.517  0.289 | 0.822  ***  *** | |
| Natural killer cell | KIR2DL1  KIR2DL3  KIR2DL4  KIR3DL1  KIR3DL2  KIR3DL3  KIR2DS4 | -0.027  0.142  0.161  -0.028  0.104  0.036  0.041 | 0.605  **  **  0.595  *  0.488  0.426 | -0.065  0.123  0.128  -0.063  0.06  0.02  0.029 | 0.225  *  *  0.243  0.27  0.705  0.588 | |
| Dendritic cell | HLA-DPB1  HLA-DQB1  HLA-DRA  HLA-DPA1  BDCA-1(CD1C)  BDCA-4(NRP1)  CD11c (ITGAX) | 0.447  0.351  0.42  0.416  0.354  0.201  0.52 | ***  ***  ***  ***  ***  ***  *** | 0.409  0.3  0.376  0.378  0.304  0.157  0.49 | ***  ***  ***  ***  ***  **  *** | |
| Th1 | T-bet (TBX21)  STAT4  STAT1  IFN-g (IFNG)  TNF-a (TNF) | 0.192  0.52  0.457  0.23  0.411 | ***  ***  ***  ***  *** | 0.124  0.489  0.435  0.195  0.379 | *  ***  ***  ***  ** | |
| Th2 | GATA3  STAT6  STAT5A  IL13 | 0.359  0.19  0.418  0.084 | ***  ***  ***  0.106 | 0.318  0.186  0.378  0.071 | ***  ***  ***  0.19 | |
| Tfh | BCL6  IL21 | 0.013  0.109 | 0.8  * | 0.011  0.104 | 0.842  0.0546 | |
| Th17 | STAT3  IL17A | 0.288  0.06 | ***  0.245 | 0.254  0.046 | ***  0.39 | |
| Treg | FOXP3  CCR8  STAT5B  TGFb (TGFB1) | 0.106  0.414  0.061  0.503 | *  ***  0.238  *** | 0.074  0.384  0.091  0.46 | 0.173  ***  0.0901  *** | |
| T cell exhaustion | PD-1 (PDCD1)  CTLA4  LAG3  TIM-3 (HAVCR2)  GZMB | 0.423  0.408  0.191  0.498  0.138 | ***  ***  ***  ***  ** | 0.381  0.36  0.136  0.471  0.077 | ***  ***  *  ***  0.155 | |

Note: *P < 0.01; **P < 0.001; ***P < 0.0001.

**Supplementary Table 3. Correlation analysis between NF-κB2 and gene biomarkers of immune cells in HCC (TIMER).**

| **Immune cells** | **Biomarkers** | None | | Purity | | |
| --- | --- | --- | --- | --- | --- | --- |
|  |  | Cor | P-value | Cor | | P-value |
| CD8+ T cell | CD8A  CD8B | 0.275  0.221 | ***  *** | 0.219  0.173 | ***  ** | |
| T cell (general) | CD3D  CD3E  CD2 | 0.336  0.353  0.351 | ***  ***  *** | 0.299  0.31  0.314 | ***  ***  *** | |
| B cell | CD19  CD79A | 0.28  0.242 | ***  *** | 0.232  0.193 | ***  *** | |
| Monocyte | CD86  CD115(CSF1R) | 0.412  0.385 | ***  *** | 0.378  0.344 | ***  *** | |
| TAM | CCL2  CD68  IL10 | 0.298  0.283  0.296 | ***  ***  *** | 0.25  0.231  0.232 | ***  ***  *** | |
| M1 Macrophage | INOS (NOS2)  IRF5  COX2(PTGS2) | 0.05  0.416  0.324 | 0.336  ***  *** | 0.029  0.418  0.28 | 0.588  ***  *** | |
| M2 Macrophage | CD163  VSIG4  MS4A4A | 0.215  0.294  0.294 | ***  ***  *** | 0.139  0.248  0.24 | **  ***  *** | |
| Neutrophils | CD66b (CEACAM8)  CD11b (ITGAM)  CCR7 | -0.018  0.512  0.304 | 0.727  ***  *** | -0.037  0.492  0.252 | 0.497  ***  *** | |
| Natural killer cell | KIR2DL1  KIR2DL3  KIR2DL4  KIR3DL1  KIR3DL2  KIR3DL3  KIR2DS4 | -0.044  0.134  0.166  -0.024  0.108  0.041  0.059 | 0.403  **  **  0.643  *  0.431  0.255 | -0.082  0.133  0.138  -0.041  0.062  0.004  0.048 | 0.129  *  *  0.449  0.25  0.943  0.378 | |
| Dendritic cell | HLA-DPB1  HLA-DQB1  HLA-DRA  HLA-DPA1  BDCA-1(CD1C)  BDCA-4(NRP1)  CD11c (ITGAX) | 0.367  0.291  0.371  0.36  0.263  0.219  0.478 | ***  ***  ***  ***  ***  ***  *** | 0.322  0.239  0.329  0.314  0.213  0.185  0.451 | ***  ***  ***  ***  ***  ***  *** | |
| Th1 | T-bet (TBX21)  STAT4  STAT1  IFN-g (IFNG)  TNF-a (TNF) | 0.157  0.399  0.367  0.206  0.385 | **  ***  ***  ***  *** | 0.093  0.371  0.342  0.177  0.364 | 0.0858  ***  ***  ***  ** | |
| Th2 | GATA3  STAT6  STAT5A  IL13 | 0.31  0.282  0.37  0.073 | ***  ***  ***  0.159 | 0.27  0.285  0.327  0.053 | ***  ***  ***  0.331 | |
| Tfh | BCL6  IL21 | 0.12  0.113 | *  * | 0.116  0.104 | *  0.0525 | |
| Th17 | STAT3  IL17A | 0.311  0.079 | ***  0.128 | 0.277  0.049 | ***  0.359 | |
| Treg | FOXP3  CCR8  STAT5B  TGFb (TGFB1) | 0.113  0.41  0.171  0.387 | *  ***  ***  *** | 0.079  0.382  0.198  0.351 | 0.142  ***  ***  *** | |
| T cell exhaustion | PD-1 (PDCD1)  CTLA4  LAG3  TIM-3 (HAVCR2)  GZMB | 0.367  0.412  0.148  0.408  0.104 | ***  ***  **  ***  * | 0.346  0.383  0.103  0.373  0.046 | ***  ***  0.056  ***  0.394 | |

Note: *P < 0.01; **P < 0.001; ***P < 0.0001.

**Supplementary Table 4. The Kinase and miRNA-target networks of RelB in HCC (LinkedOmics).**

| \| **Enriched Category** \| \| --- \| | **Geneset** | **LeadingEdgeNum** | **P-value** |
| --- | --- | --- | --- | --- |
| **Kinase Target**  **miRNA Target** | Kinase_ SYK  Kinase_ LCK  Kinase_ PRKCG  Kinase_ LYN  Kinase_ ROCK1  ATAAGCT, MIR-21  TAATGTG, MIR-323  ATGTTAA, MIR-302C  TAGGTCA, MIR-192, MIR-215  TCTATGA, MIR-376A,MIR-376B | 19  22  18  25  13  38  50  72  15  25 | 0  0  0  0  0  0  0  0  0  0 |

**Table 5. The Kinase and miRNA-target networks of NF-κB2 in HCC (LinkedOmics).**

| \| **Enriched Category** \| \| --- \| | **Geneset** | **LeadingEdgeNum** | **P-value** |
| --- | --- | --- | --- | --- |
| **Kinase Target**  **miRNA Target** | Kinase_ CHUK  Kinase_ PPKDC  Kinase_ IKBKB  Kinase_ ROCK1  Kinase_ PPKCA  ACACTGG, MIR-199A,MIR-199B  GCATTTG, MIR-105  TAGCTTT, MIR-9  TGCACTT, MIR-519C,MIR-519B,MIR-519A  GCTTGAA, MIR-498 | 8  6  10  15  68  41  57  69  139  37 | 0  0  0  0  0  0  0  0  0  0 |
